# Immediate Effects of Ammonia Shock on Transcription and Composition of a Biogas Reactor Microbiome

**DOI:** 10.3389/fmicb.2019.02064

**Published:** 2019-09-06

**Authors:** Martin A. Fischer, Andrea Ulbricht, Sven C. Neulinger, Sarah Refai, Kati Waßmann, Sven Künzel, Ruth A. Schmitz

**Affiliations:** ^1^Department of Biology, Institute of General Microbiology, Christian-Albrechts-University Kiel, Kiel, Germany; ^2^Department of Biology, Institut für Mikrobiologie und Biotechnologie, University Bonn, Bonn, Germany; ^3^Department for Evolutionary Genetics, Max-Planck-Institute for Evolutionary Biology, Plön, Germany

**Keywords:** anaerobic digestion, biogas, ammonia, next-generation sequencing, 16S microbial community structure, metatranscriptomics

## Abstract

The biotechnological process of biogas production from organic material is carried out by a diverse microbial community under anaerobic conditions. However, the complex and sensitive microbial network present in anaerobic degradation of organic material can be disturbed by increased ammonia concentration introduced into the system by protein-rich substrates and imbalanced feeding. Here, we report on a simulated increase of ammonia concentration in a fed batch lab-scale biogas reactor experiment. Two treatment conditions were used simulating total ammonia nitrogen concentrations of 4.9 and 8.0 g/L with four replicate reactors. Each reactor was monitored concerning methane generation and microbial composition using 16S rRNA gene amplicon sequencing, while the transcriptional activity of the overall process was investigated by metatranscriptomic analysis. This allowed investigating the response of the microbial community in terms of species composition and transcriptional activity to a rapid upshift to high ammonia conditions. Clostridia and Methanomicrobiales dominated the microbial community throughout the entire experiment under both experimental conditions, while Methanosarcinales were only present in minor abundance. Transcription analysis demonstrated clostridial dominance with respect to genes encoding for enzymes of the hydrolysis step (cellulase, EC 3.2.1.4) as well as dominance of key genes for enzymes of the methanogenic pathway (methyl-CoM reductase, EC 2.8.4.1; heterodisulfide reductase, EC 1.8.98.1). Upon ammonia shock, the selected marker genes showed significant changes in transcriptional activity. Cellulose hydrolysis as well as methanogenesis were significantly reduced at high ammonia concentrations as indicated by reduced transcription levels of the corresponding genes. Based on these experiments we concluded that, apart from the methanogenic archaea, hydrolytic cellulose-degrading microorganisms are negatively affected by high ammonia concentrations. Further, *Acholeplasma* and Erysipelotrichia showed lower abundance under increased ammonia concentrations and thus might serve as indicator species for an earlier detection in order to counteract against ammonia crises.

## Introduction

Anaerobic degradation of organic biomass, such as agricultural waste or maize silage, for the production of biogas is of increasing importance in terms of providing renewable and flexible green energy (Weiland, 2010). The production of biogas consists of a four-step process of hydrolysis, acidogenesis, acetogenesis, and methanogenesis. While the first three steps are mostly performed by the bacterial and, to a lesser extent, eukaryotic community (Bengelsdorf et al., 2013), the final step of methane production is a unique trait of methanogenic archaea (Offre et al., 2013). Correlation of disturbances in biogas processes and microbial community composition have been in the spotlight of research for over a decade (Chachkhiani et al., 2004). Microbial community composition and the activity of its members were shown to correlate with factors like substrates (Francisci et al., 2015; Alsouleman et al., 2016), pH (Lu et al., 2013; Goux et al., 2015), temperature (De Vrieze et al., 2015), and fermentation conditions (Stolze et al., 2015) during the degradation process. Firmicutes, Bacteroidia, and Proteobacteria were in many cases the most abundant bacterial taxa (Li et al., 2013; Stolze et al., 2015; Güllert et al., 2016; Luo et al., 2016) whereas the methanogenic archaeal community was observed to be less diverse compared to the bacterial domain (Francisci et al., 2015). Methanosaetaceae and Methanosarcinaceae have been frequently observed in anaerobic degradation where they contribute to methane formation mainly *via* the acetoclastic pathway (Karakashev et al., 2006; Fotidis et al., 2013). On the other hand, Methanomicrobiales and Methanobacteriales dominate in biogas reactors with a predominance of hydrogenotrophic methanogenesis (Fotidis et al., 2014; Stolze et al., 2015; Alsouleman et al., 2016; Luo et al., 2016). Today, advanced research techniques allow a more detailed insight into composition and function of the community involved in the degradation process than ever before.

Especially the response of the microbial community to reactor malfunction or process inhibition is crucial for a better understanding of microbial regulation for optimizing process stability and productivity (Niu et al., 2013; Lv et al., 2014). Several factors causing process inhibition have been identified, such as high hydrogen sulfide or ammonia concentrations, light or heavy metals, as well as the presence of certain organic substances like halogenated alkanes or aromatics (reviewed in Yenigün and Demirel, 2013). Among these, process inhibition by increased ammonia concentration is one of the most frequent incident types (Chen et al., 2008; Yenigün and Demirel, 2013). Inhibitory concentrations of ammonia in biogas reactors were reported on a wide range between 600 and 14,000 mg/L total ammonia nitrogen (TAN) (Chen et al., 2008, 2016; Yenigün and Demirel, 2013). This wide range results from the individual microbial community and process parameters of each individual biogas reactor. Acclimatization experiments provided evidence that the threshold for inhibition can be increased by proper adaptation of the microbial community (Van Velsen, 1979; Westerholm et al., 2011; Fotidis et al., 2013; Gao et al., 2015). The toxicity of ammonia seems to be primarily caused by the dissociated form (NH_3_). The parameters influencing the equilibrium between ${{NH}_{4}}^{+}$ and NH_3_ such as pH and temperature therefore additionally influence the inhibitory potential of the TAN concentration (Gallert et al., 1998). High ammonia concentrations are toxic to microbial cells in two ways: first under increased ammonia conditions, uncharged ammonia molecules may passively diffuse into the microbial cell through the membrane leading to a pH imbalance of the cell interior and inhibiting enzymes involved in the metabolism (Gallert et al., 1998; Chen et al., 2008). Secondly, loss of intracellular NH_3_ by diffusion through the cytoplasmic membrane after previous permease-mediated uptake as ${{NH}_{4}}^{+}$ could result in decreasing the proton motive force (Neijssel et al., 1990; Kadam and Boone, 1996; Chen et al., 2008). In a full-scale biogas reactor, ammonium can be introduced into the system by nitrogen- and protein-rich feedstock such as slaughter house waste and poultry manure (Chen et al., 2008; Acs et al., 2013; Zhang et al., 2014). These substrates are economically and ecologically interesting for the production of biogas as they are waste or side products and have a high bio-methane potential (Kovács et al., 2013; Moestedt et al., 2016). During anaerobic degradation, the protein portion of these substrates gets hydrolyzed into peptides and single amino acids. The release of ammonium from the amino acids occurs during transamination and deamination processes (Kovács et al., 2013). Biogas reactors show a broad range of concentrations from 1.7 to 9 g/L TAN with stable methane production after community adaptation (Angelidaki and Ahring, 1993; Yenigün and Demirel, 2013). Especially the methanogenic community appeared to be very sensitive toward changes in ammonia conditions. Among the methanogens, acetoclastic methanogenesis was observed to be inhibited already at lower ammonia levels whereas hydrogenotrophic methanogenesis was less sensitive toward increased ammonia concentrations (Sprott and Patel, 1986; Schnürer et al., 1994; Fotidis et al., 2014; Fischer et al., 2018).

Aiming to investigate the immediate response of the microbial community, its composition and gene expression in response to a rapid ${{NH}_{4}}^{+}$ upshift, we applied multiple analysis techniques in a replicated lab-scale simulation of a drastic increase in ammonium concentration from 4.9 to 8.0 g/L TAN using an inoculum from a previously described commercial biogas reactor. This reactor and its microbial community were already adapted to increased ammonium concentrations of 4.9 g/L TAN (Fischer et al., 2018). Methane production was used to monitor overall microbial metabolic productivity during the experiment. Microbial community composition was analyzed by 16S rRNA gene amplicon sequencing of the bacterial and archaeal community on a daily sampling basis. Moreover, metatranscriptome analysis was applied at defined time points to determine the active community and transcript levels of enzymes involved in the anaerobic degradation process.

## Materials and Methods

### Reactor Setup and Incubation

The starting material was obtained from a recently described reactor (Fischer et al., 2018). The volume of the commercial biogas fermenter was 2,800 m^3^, the organic load rate was 4.1 kg of organic dry matter per m^3^ per day (3.7–4.5 within a year before and after sampling), and the hydraulic retention time was 76.1 days (varied between 69 and 93 days within a year before and after sampling). The methane production was 2.36 ± 0.24 μmol/g·h (1.1–3.3 μmol/g·h within a year before and after sampling), and the pH of the biogas reactor was 8.1 (7.9–8.1 within a year before and after sampling). The conductivity of the biogas sludge was determined to be 45.4 mS/cm. Volatile fatty acid concentration of the starting material was 5.36 g/L (acetic acid equivalents), the total alkalinity of carbonate was 25.7 g CaCO_3_/L. The dry weight and organic dry weight of the biogas sludge were measured to be 109 and 79.6 g/kg respectively. Primary feeding consisted of maize silage, cattle manure, and poultry dry manure. The reactor operated at 40°C. Average power production in the sampling month was 531 kW/h. Methane produced from fresh biogas reactor material was 2.37 μmol/g·h. Physicochemical parameters were measured in the sampling month by the operator in accordance to VDLUFA (Janßen, 2011). Samples were collected in sealed plastic bottles transported and stored at 4°C until setup of the lab-scale reactors.

Eight small-scale fed batch reactors used in the experiment were set up as previously described by Refai et al. (2014a,b). Briefly, 200 g of biogas reactor material was transferred under anaerobic conditions to 1-L gas-tight glass bottles sealed with butyl rubber stoppers and locked with aluminum screw caps. Ammonium was added to the ammonia treatment reactors as 5 M NH_4_Cl solution to reach a final concentration of 8 g/L TAN. TAN concentration of the control reactors remained unchanged at 4.9 g/L TAN. Cultures were subsequently treated with N_2_/CO_2_ (50/50) for 10 min and incubated at 40°C on an incubation shaker. Biogas formation was measured daily as described below over a period of 10 days. Two grams of sample material were taken every day under anaerobic conditions and directly stored at −80°C until nucleic acid isolation and determination of the volatile fatty acid (VFA), acetate, and butyrate concentration. Reactors were fed with premixed and shredded substrate, resembling the composition of the feed used in the original biogas reactor and consisting of (dry weight) maize silage (42.7%), cow manure (37.4%), and dried poultry manure (19.9%). Also, 2.5 g of this mix as well as 0.4 ml of recirculate obtained from the original biogas reactor material were added to the biogas reactor every sampling day, as previously described (Refai et al., 2014b). After sampling and feeding, the headspace of the reactors was flushed with N_2_/CO_2_ (50/50) for 10 min, and incubation was continued at 40°C.

### Measurement of Biogas Production and Methane Content

Biogas production for the individual biogas reactors was measured daily with exception of day 8 by correlation of biogas volume and concentration of CH_4_ in the headspace of the reactors. The volume of the produced biogas was determined using a gas tight tube connected to an immersed volumetric cylinder. For measuring the CH_4_ concentration, 30-μl samples from the headspace of the reactor were analyzed by gas chromatography (GC, Perkin Elmer Clarus® 480, Rascon FFAP column 25 m 0.25 μm, Perkin Elmer, Waltham, USA) with an FID. GC parameters were set to a column temperature of 120°C, an injector temperature of 150°C, and a detector temperature of 250°C with N_2_ as carrier gas. Ten percent methane standard (90% Ar, Air Liquid, Düsseldorf, Germany) was analyzed before and after every series of measurements and used for calculation of methane yield by correlation. The results were normalized to standard conditions and methane formation rates were calculated with the specified unit of micromoles of CH_4_ per gram sludge per hour (μmol/g·h).

### Analysis of Acetate and Butyrate Concentrations

Due to low sampling material amounts, the replicated samples from the four individual reactors per treatment condition and sampling point were combined. Acetate and butyrate concentrations were determined by gas chromatography as previously described (Refai et al., 2014a).

### Nucleic Acid Extraction

DNA was extracted from ~300 mg reactor sample using the NucleoSpin Soil Kit (Machery-Nagel, Düren, Germany) following the instructions of the manufacturer. Extracted DNA was stored at −20°C.

RNA was extracted from 0.5 g of reactor sample. Samples were frozen in liquid N_2_ and homogenized using an dismembrator instrument (Sartorius AG, Göttingen, Germany) with 2000 rpm for 5 min. Samples were transferred into 2.5-ml Isoyl-RNA Lysis reagent (5 Prime GmbH, Hilden, Germany) and RNA was extracted applying the Direct-zol RNA Kit (Zymo Research, Freiburg, Germany). The protocol for RNA purification included on-column DNase I treatment (5 μl, 6 U/μl, Zymo Research, Freiburg, Germany) according to manufacturer instruction. Extracted RNA was stored at −80°C until further preparation.

### PCR and Amplicon Sequencing

Extracted DNA was adjusted to a concentration of 20 ng/μl and applied in the amplification reaction. Primers applied for the amplification of the bacterial and archaeal 16S rRNA gene fragments are listed in Supplementary Table S1 and were previously tested for applicability to monitor the microbial community in biogas environments (Fischer et al., 2016). Primers consisted of a target region-specific fraction, an Illumina linker region, a barcode, and an Illumina P5 or P7 region. The PCR mix contained: 12.6 μl of H_2_O (Roth), 0.4 μl of 10 mM dNTPs (Thermo Fisher Scientific), 4 μl of 5× Phusion HF-buffer (Thermo Fisher Scientific), 0.8 μl of 5 μM primers (MWG and Biomers), 0.2 μl of Phusion high-fidelity polymerase (2 U/μl, Thermo Fisher Scientific), and 2 μl of extracted DNA. Cycling conditions for PCR amplification started with an initial denaturation step for 30 s at 95°C, followed by 30 cycles of 10 s at 95°C, 45 s at 52°C for the bacterial 16S and 56°C for the archaeal 16S primer pair and 30 s at 72°C. The final extension step was set to 10 min at 72°C. All reactions were performed with one corresponding negative control containing additional 2 μl of H_2_O instead of DNA extract. Amplicons were checked for correct length and contamination in the negative control *via* agarose gel electrophoresis. Bands with correct length were sliced out of the gel and purified using the MinElute Gel Extraction Kit (Qiagen, Hilden, Germany). DNA concentration in eluates ranged from 10 to 118 ng/μl, as quantified fluorospectrometrically using the Quant-iT Kit. Eluates were pooled equimolarly.

Libraries were prepared according to the manufacturer’s instructions and sequenced on a MiSeq instrument (Illumina Inc., USA, CA, San Diego) using the v3 chemistry with 2 × 300 bp paired-end. The numbers of the individual reads per sample are summerized in Supplementary Table S4. Sequences were submitted to the NCBI sequence read archive and are accessible under BioProject PRJNA315559 (SRX3323391 to SRX3323566).

### Processing and Analysis of 16S Amplicon Dataset

Sequencing reads were trimmed using the trimmomatic software version 0.33 (Bolger et al., 2014). Briefly, reads were analyzed with a sliding window of 4 bp. Regions were trimmed if the average Phred score (Ewing et al., 1998; Ewing and Green, 1998) within the window was below 30. Illumina adapters and primer sequences were removed. Clean reads were kept within the dataset if the forward and reverse reads both survived the quality trimming and were longer than 36 bp.

Quality-trimmed sequences were analyzed using MOTHUR software, version 1.35.1 (Schloss et al., 2009). The analysis of the reads was performed as described recently (Fischer et al., 2018). Sequences were taxonomically classified using the Greengenes database (Version 13_05_99) (DeSantis et al., 2006). Information regarding the sequences generated and used for the 16S amplicon analysis is summarized in Supplementary Tables S2, S3.

Analysis and visualization were carried out using R version 3.2.4 (Team, 2015) as well as the packages vegan (Oksanen et al., 2015), Hmisc (Harrell and Dupont, 2008), dichromat (Lumley, 2013), and scales (Wickham, 2012). Absolute abundance of OTUs generated at the 97% similarity level was transformed using Hellinger transformation (Legendre and Gallagher, 2001) by application of Eq. (1).

(1) $$y_{ij}^{\prime }=\sqrt{\frac{y_{ij}}{y_{j+}}}$$

where *y_ij_* is the abundance of OTU *i* in sample *j* and *y*_*j*+_ is the cumulative OTU abundance in sample *j*, and $y_{ij}^{\prime }$ is the transformed abundance of OTU *i* in sample *j*.

Redundancy analysis (RDA) was performed to explore the change in OTU composition with treatment (explanatory variable “Ammonia”) over time (explanatory variable “Day”) based on the Hellinger-transformed count data. Analysis of variance (ANOVA) was conducted with 1,000 permutations to test if treatment and time effects as well as their interaction were statistically significant.

### Metatranscriptome Analysis

Samples were taken from all (four) reactors of the control and all (four) reactors of the treatment group at day 3 as well as at day 6 summing up to 16 individual samples. TruSeq reagent Kit, including degradation of ribosomal RNA according to manufacturer’s protocol using the RiboZero (Illumina Inc., USA, CA, San Diego) was used for library preparation and sequencing. Sequencing of RNA from the reactor units was performed on a NextSeq Instrument (Illumina Inc., USA, CA, San Diego) using the NextSeq 500 Mid Output Kit for 300 cycles according to the manufacturer’s protocol.

The generated raw sequences were trimmed using the software trimgalore version 0.3.7 (Krüger, 2012) in paired end mode including adapter trimming, quality check for a Phred value above 30 (Ewing et al., 1998; Ewing and Green, 1998), and a minimum sequence length of 20 bp. Quality of remaining reads was assessed with fastQC (Andrews, 2010). MegaHit version 1.0.2 (Li et al., 2015) was used for the combined assembly of all reads generated for the different time points as well as the publicly available metagenome from the corresponding biogas reactor (46) using a succession of k-mer length of 21, 41, 61, 81, 91, and 99. Contigs with a length below 300 bp or coverage under 6 were omitted from the assembly. A total of 404,069 contigs were assembled during the process containing overall 307,529,181 bp. The longest contig contained 28,714 bp, N50 value of the assembly was 900 bp. Backmapping of individual reads was performed with BBmap version 35.37 (Bushnell, 2014) to calculate read recruitment and contig coverage for the individual sample datasets; 60% of the clean reads could be mapped back onto contigs. Samtools was used for sorting the mapped reads. RNAhmm3 (Lagesen et al., 2007) was used for removal of ribosomal RNA sequences. Open reading frames (ORFs) of ≥80 codons in length were predicted with MetaProdigal version 2.6.2 (Hyatt et al., 2010). ORFs were assigned to their potential function using Interproscan version 5.14-33 (Quevillon et al., 2005) and the Pfam (version 28) (Bateman et al., 2004), TIGRfam (version 15) (Haft et al., 2003) and Superfamily (version 1.75) (Madera et al., 2004; Wilson et al., 2006) databases. HTSeq (Anders et al., 2015) was used for calculation of read recruitment on predicted genes. Megablast (Altschul et al., 1990, 1997) was used for taxonomic annotation of the contigs using the nt reference databases (Sayers et al., 2009). After initial annotation, TAMER (Jiang et al., 2012) was used to resolve conflicts with multiple best reference hits. Results of the different modules were combined into a PostgreSQL database using an in-house R script. Information regarding the statistics of the assembly is summarized in Table 1.

**Table 1:** Core statistics regarding the assembled metatranscriptome.

Number of unfiltered scaffolds (>0 nt)	404,069
Number of scaffolds (>300 nt)	217,077
Total size of scaffolds	214,707,994 nt
Longest scaffold	28,714 nt
Shortest scaffold	300 nt
Number of scaffolds >1K nt	59,565
Number of scaffolds >10K nt	423
Mean scaffold size	989 nt
Median scaffold size	586 nt
N50 scaffold length	1,414 nt
L50 scaffold count	37,998
GC content	42.54%
Predicted ORFs	194,119

*All information on scaffolds refer to the filtered scaffolds (>300 nt)*.

ORFs of interest were extracted from the dataset using their respective Enzyme commission reference number (E.C. number). As an additional filter step, reads not annotated as bacterial or archaeal were removed from the dataset, since the focus was set on the microbial community. The E.C. read counts of the respective genes were normalized to absolute abundance within their corresponding dataset.

Statistical analysis was done applying the two-sided Wilcoxon rank sum test performed in R 3.2.4 (Team, 2015). Treatment condition (ammonia-shocked and control) and sampling time point (days 3 and 6) were used for defining replicate groups. Results were considered statistically significant when the *p* ≤ 0.05.

Taxonomic origin of the transcripts was investigated in each gene of interest by extracting the annotation of the contig containing the respective ORF. The obtained community composition was analyzed on the order level using R 3.2.4 (Team, 2015). For visualization of the main differences in community composition, the data were square-root transformed and principal component analysis was applied. Results were visualized using the R-script ggbiplot (Wickham and Chang, 2008).

## Results and Discussion

### Reactor Performance Shows Drastic Decrease of Methane Formation Under High Ammonia Levels

**Figure 1:**
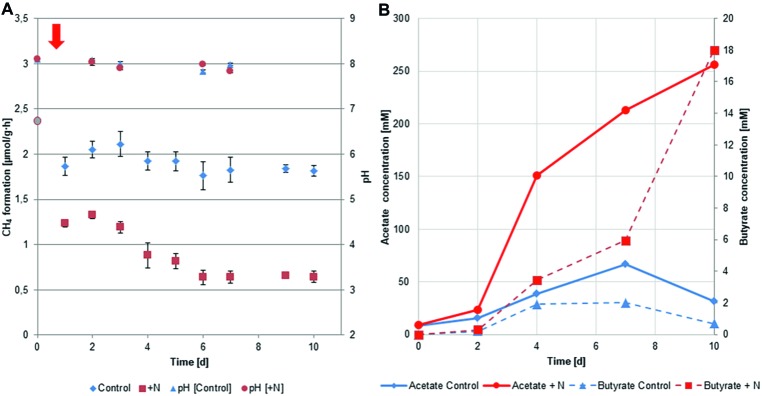
**(A)** Methane formation and pH during the experiment within the control and ammonia-treated reactors (*n* = 4). Addition of ammonia was performed at the start of the experiment (day 0) symbolized by the red arrow. Methane formation for day 0 (**•**) was measured from the reactor material used to setup the reactors. **(B)** Concentrations of acetate and butyrate observed within the course of the experiment in the two reactor setups. Measurements refer to the combined replicates (four reactors) per treatment.

Within the experiment, methane (CH_4_) production was measured every day as described in the method section. Performance of the biogas reactors in terms of CH_4_ production is illustrated in Figure 1A. Methane production of the fresh biogas reactor material was determined before the setup of the experiment and was 2.37 ± 0.35 μmol per gram of wet biogas reactor material per hour (μmol/g·h). CH_4_ production was significantly lower at all individual time points in reactors with increased ammonia concentration (average production during experiment 0.9 ± 0.3 μmol/g·h) compared to control fermenters (Wilcoxon rank sum test; *W* = 16, *p* < 0.03). Reactor performance of the control group (average production during experiment 1.9 ± 0.1 μmol/g·h) was comparable to previous studies under similar conditions (Refai et al., 2014b).

VFAs are key intermediates in the anaerobic degradation and their concentrations are important parameters in monitoring the state of biogas reactors (Hansen et al., 1998; Weiland, 2010; Lv et al., 2014). The individual degradation of VFAs such as acetate, butyrate, and propionate under different conditions has been in the focus of different studies, as their metabolization can give important information on the fitness of the microbial community (Refai et al., 2017). A clear difference was observed in the acetate and butyrate concentrations between the two setups (Figure 1B). While in the control group, acetate (8.5–66.4 mM) and butyrate (0–2.02 mM) concentrations remained on a moderate level they accumulated in the treatment reactors up to 256.5 mM and 18 mM respectively during the course of the experiment. These findings indicate that between day 2 and 4, acetate already started to accumulate in the ammonia-treated fermenters due to the inhibition of the methanogenesis. Butyrate levels at this point were still comparable between the two setups, pointing toward a still ongoing degradation of butyrate. Butyrate level at day 7 and 10 however show clear differences between the two setups and hint toward a potential beginning acidification or the beginning establishment of an “inhibited steady-state” (Angelidaki and Ahring, 1993) in the ammonia-treated reactors.

Besides physiochemical parameters such as VFA concentrations (Moestedt et al., 2016) and the ability to degrade intermediates (Refai et al., 2017), CH_4_ formation can be used to monitor the productivity of a reactor (Ziganshin et al., 2013; Refai et al., 2017) as it is a final product of the anaerobic degradation. Thus, lower CH_4_ yield points to a less effective degradation and possibly toward an imbalanced system, in our case as a result of increased ammonia concentration. This observed effect is in agreement with production-scale biogas reactors influenced by increased ammonia concentrations (Chen et al., 2008). During the time of the experiment, all other incubation parameters such as temperature, pH, mixing, and feeding were kept constant and identical for the ammonium-shocked and control reactors. In other studies, the progressive increase in ammonia concentration and its monitoring over prolonged periods of time has been investigated by the addition of protein-rich feedstock (Ziganshin et al., 2013; Lv et al., 2014; Kovacs et al., 2015; Bonk et al., 2018). In these experiments, ammonia is released by the degradation of the substrate gradually and simulates a rather natural process closer to the one occurring in the commercial-scale biogas reactor. The addition of ammonia as adjusted solution was alternatively applied in other studies (Fotidis et al., 2013; Dai et al., 2016; Bonk et al., 2018) and allows for the precise definition of concentration and starting point of the experiments necessary for the proceeding analysis within the replicated setup applied here.

### 16S rRNA Gene Microbial Community Analysis Identifies Clostridia, Bacteroidia, and Methanomicrobiaceae as Dominant Taxa

**Figure 2:**
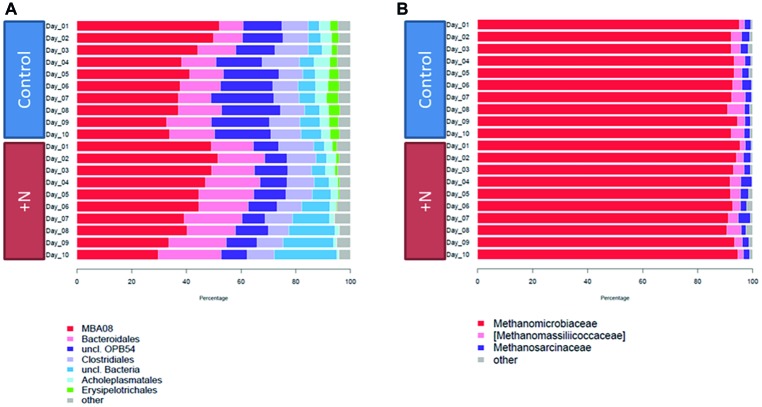
Bacterial **(A)** and archaeal **(B)** community composition over time on the family level. Taxa with abundance below 2% were grouped into “others.”

Bacterial community composition within the biogas reactors was monitored *via* 16S rRNA gene amplicon sequencing. All experimental reactors were prepared from the same biogas reactor sample, hence ammonia-treated and control reactors contained an identical community structure at the start of the incubation. However, in response to the addition of ammonium, community composition clearly changed over time (Figure 2).

Concluding from microbial 16S rRNA gene amplicon composition, Clostridia and Bacteroidia were the most abundant classes during the experiment in both setups. The proportion of Clostridia in the control and ammonia-treated fermenters on day 1 was ~63% and decreased over the 10 days of incubation to ~46% in the control and to ~40% in the ammonia-treated reactors (Figure 2A). Taxonomic analysis of the provisional order-level taxon showed that MBA08 and the family Caldicoprobacteraceae were the most abundant clostridial taxa in the dataset. The diverse MBA08 group already has been observed in thermophilic biogas reactors (Cheon et al., 2007; De Vrieze et al., 2015) where they largely contribute to cellulose degradation (Lynd et al., 2002). This taxon is suspected to contain a large number of closely related eurythermal species (Sun et al., 2016).

In the present experiment, MBA08 accounted for 30–52% of the composition of bacterial sequences. The Caldicoprobacteraceae accounted for 6–11% of the bacterial community. Both bacterial families were slightly decreasing in abundance during the course of the experiment while remaining the most abundant families within the Clostridia. With regard to the treatment conditions, the abundance of both MBA08 as well as Caldicoprobacteraceae did not show significant differences between the treatment and control reactors. Both families were recently observed in a study investigating model biogas reactors showing increased ammonia concentrations due to nitrogen-rich substrates (Lv et al., 2019). This study showed that especially Caldicoprobacteraceae increased in abundance under partial or complete inhibition due to high ammonia conditions and largely contributed to the observed variance between inhibited and non-inhibited reactors (Lv et al., 2019). In other biogas reactor setups, they were observed to be abundant under alternating temperature conditions in biogas reactors (Sun et al., 2015) and were particularly abundant under increased ammonia conditions (Müller et al., 2016; Poirier et al., 2016). Since the used starting biogas reactor material in our study was already showing increased TAN concentrations of 4.9 g/L, our observation of the high abundance of the MBA08 as well as the Caldicoprobacteraceae further underlines their association with increased ammonia conditions.

The second most abundant class was Bacteroidia, showing an abundance of 8% in the control and 15% in the ammonia-treated reactors after 1 day of incubation. After 10 days of incubation, Bacteroidia abundances increased to 16% in the control and 23% in the ammonia-treated reactors. The family Porphyromonadaceae, often found in biogas reactor samples (Hahnke et al., 2015; Müller et al., 2016), dominated this class. Members of this family contain enzymes for degradation of a wide range of organic substrates such as proteins, carbohydrates, and polysaccharides (Krieg et al., 2001; Chen and Dong, 2005; Ziganshin et al., 2011; Hahnke et al., 2015). Apart from Porphyromonadaceae, the genus-level taxon BF311 (Bacteroidetes) was also abundant within the class Bacteroidia. This taxon was recently observed in the cow rumen (Kumar et al., 2015) and in lab-scale reactor experiments where its abundance positively correlated with increased nitrate concentration (Zhao et al., 2015).

OPB54 (Firmicutes) in control reactors accounted for 14% of the bacterial community at the start and 21% at the end of the experiment. In the ammonia-treated reactors, their abundance stabilized during the experiment at around 9% of the bacterial community. Representatives of this class were initially found in 16S rRNA gene sequences recovered from hot springs in Yellow Stone National Park (Hugenholtz et al., 1998) and have also been observed recently in biogas reactor samples, particularly abundant under increased loading rate and reactor acidification (Roske et al., 2014). A first isolate of this candidate taxon was obtained from a mesophilic anaerobic sludge reactor treating herbicide waste. It was characterized as a strictly anaerobic, spore-forming, hydrogen-producing bacterium. This isolate, *Hydrogenispora ethanolica*, was observed to produce H_2_, ethanol, and acetate by fermentation of various saccharides (Liu et al., 2014).

At the beginning of the incubation experiment, Erysipelotrichia and Mollicutes were almost equally low in abundance in the control (3 and 4%, respectively) and treatment reactors (2 and 3%, respectively). While their abundance remained relatively stable under control conditions, it steadily decreased over time under increased ammonia conditions. After 10 days of incubation, Erysipelotrichia still accounted for 3% of the sequences in the control reactors, while their abundance decreased to 0.1% in the ammonia-treated reactors. The most abundant family within Erysipelotrichia were Erysipelotrichaceae, and within them the uncharacterized bacterium RFN20. Information regarding their potential contribution to the anaerobic degradation is scarce since no genome information is currently available. They have been observed as part of a biogas reactor microbiome stably operating under increased ammonia conditions and positively correlated with increased ammonia concentrations (Müller et al., 2016). Interestingly, 16S rRNA gene sequences of RFN20 have been identified in a mouse gut microbiome study where they appeared to positively correlate with diet fat ratio, but could not be linked to a specific function within the community (Fleissner et al., 2010).

Mollicutes accounted for 3% of the sequences at the end of the experiment in the control reactors, while their abundance in the ammonia reactors was only 1%. The genus *Acholeplasma* was the main representative of this class. This genus is frequently observed in biogas reactors (Krober et al., 2009; Solli et al., 2014; Bengelsdorf et al., 2015) and harbors enzymes for degradation of a variety of saccharides (Freundt et al., 1984; Razin, 2006). In a study by Müller et al. (2016), *Acholeplasma* increased in abundance under increasing ammonia concentrations and a higher proportion of syntrophic acetate oxidation (Müller et al., 2016). In addition to already classified taxa, we observed a considerable proportion of unclassified bacterial sequences (4–23% in the ammonia-treated reactors and 7% in the control reactors) which points toward the importance of and requirement for ongoing isolation and characterization of microbes from this complex environment (Kern et al., 2016; Maus et al., 2016a).

The observed dominance of the phylum Firmicutes, including the classes Clostridia, OPB54, Mollicutes, and Erysipelotrichia, in biogas reactors has been previously reported in manure-based mesophilic and thermophilic reactors with TAN concentrations ranging from 2.4 to 4.2 g/L (Luo et al., 2016). Firmicutes, especially Clostridia, are known as degraders of cellulose and other vegetable polymers. They are involved in the process of hydrolysis, acido- and acetogenesis (Wirth et al., 2012). Bacteroidia are also known cellulose degraders and often found in cellulolytic habitats like the cow rumen (Naas et al., 2014; Henderson et al., 2015; Güllert et al., 2016), the termite gut (Kohler et al., 2012), or in biogas reactors (Güllert et al., 2016).

Most archaeal sequences were assigned to the class Methanomicrobia, and almost exclusively to genus *Methanoculleus,* with abundances of over 90% in all reactors throughout the experiment (Figure 2B). This dominating abundance of *Methanoculleus* in biogas reactor samples has been reported in several studies and partially correlated with increased ammonia concentrations already present in the starting material of the experiment (4.9 g/L TAN) (Stolze et al., 2015; Güllert et al., 2016; Moestedt et al., 2016; Maus et al., 2016b; Fischer et al., 2018). Their predominance under high ammonia conditions points toward a mainly hydrogenotrophic pathway in the methanogenesis which is discussed in more detail in the following section on transcriptional analysis. Briefly, under moderate ammonia concentrations and in the presence of acetoclastic methanogens (e.g., Methanosarcinaceae or Methanosaetaceae), acetate can be converted to methane *via* acetoclastic methanogenesis, whereas under increased ammonia concentration, the conversion is bypassed *via* syntrophic acetate oxidation coupled to hydrogenotrophic methanogenesis.

Besides the dominant Methanomicrobiales, two families belonging to the Methanoplasmatales and the Methanosarcinales were detected in noticeable percentage for the duration of the incubations. Methanomassiliicoccaceae contributed between 2 and 6% of the analyzed sequences. Archaea of this family have been originally isolated from the termite intestine (Paul et al., 2012) and the human gut (Dridi et al., 2012; Borrel et al., 2016) and additionally identified in continuously stirred tank lab-scale biogas reactors fed with a protein-rich substrate and TAN concentrations up to 6 g/L as well as full-scale reactors with maize silage as main substrate with a TAN concentration of around 1 g/L (Acs et al., 2013; Lucas et al., 2015). They were also observed in treatment reactors with increased organic load rate (6 g of volatile solids per L per day) and increased ammonia concentrations of 6.9–7.5 g/L TAN (Moestedt et al., 2016) as well as in ammonia-inhibited model biogas reactors, where they were found in high abundance (44% of *mcrA* transcripts) at up to 10 g/L TAN (Lv et al., 2019). Methanomassiliicoccaceae are capable of utilizing methanol and methylamines in the presence of H_2_ for methanogenesis and potentially harbor a unique heterodisulfide reductase system (Kroninger et al., 2016). In addition, *Methanomicrococcus*, a genus belonging to the Methanosarcinales and also capable of methane formation from methanol, methylamines, and H_2_ (Sprenger et al., 2005), was present within the dataset with abundances between 2 and 3%. Sequences originating from methanogens like *Methanosarcina* and *Methanomassiliicoccus* were of consistently lower abundance for the duration of the experiment.

The changes and variation as well as the overall observed alpha diversity in the archaeal dataset were much lower compared to the diversity in bacterial community [Median Shannon alpha diversity of 1.43 (Archaea) vs. 11.85 (Bacteria)]. This lower diversity in archaea was previously described for example in thermophilic lab-scale biogas reactors with TAN concentrations between 2.1 and 3.3 g/L (Francisci et al., 2015). It is consistent with the fact that substrates of methanogenesis are much less diverse and including CO_2_, H_2_, formate, acetate, methanol, and methylated compounds (Thauer et al., 2008; Klang et al., 2015). The bacterial community, however, is involved in hydrolysis, acidogenesis, and acetogenesis using a variety of substrates. This high substrate diversity bears the potential of multiple pathways which require multiple species to break down and degrade substrates such as starch, lignin, cellulose, proteins, fatty acids, and a variety of intermediates.

In summary, a typical microbial community of a biogas reactor characterized by increased ammonia concentrations and a dominating hydrogenotrophic methanogenesis pathway was observed within the control samples. In response to additional drastic increased ammonia concentrations, the microbial community structure changed in the course of the experiment. A high percentage of Clostridia and Bacteroidia and the abundance of Methanomicrobiaceae as dominant family within the archaeal domain are in agreement with already observed full-scale biogas reactors (Westerholm et al., 2012; Müller et al., 2016; Fischer et al., 2018). In a comparable study focusing on the bacteria involved in syntrophic acetate oxidation (SAO), an even more severe community shift over an extended period of time was observed under increased ammonia conditions (5.5–6.9 g/L) (Müller et al., 2016). These results show the comparability of the applied laboratory model system when monitoring the effects of ammonia on community composition in bioreactors.

### Beta Diversity Analysis Showed the Response of the Microbiome to Treatment Conditions

**Figure 3:**
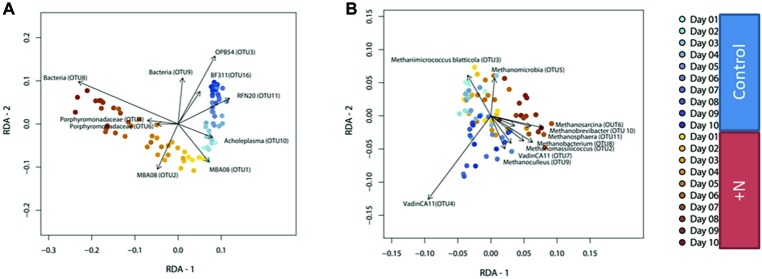
RDA based on Hellinger-transformed OTU-count data of the bacterial **(A)** and archaeal **(B)** community. Clear diversion between the two treatment types (ammonia and control) increasing over time can be observed. Samples belonging to the control group are colored in blue, ammonia-treated samples are colored yellow to orange.

Changes in community composition observed through 16S rRNA gene sequences were investigated with redundancy analysis (RDA) (Borcard et al., 2011; Legendre and Legendre, 2012; Stratil et al., 2013). The analysis was conducted with Hellinger-transformed OTU-count data with the explanatory variables ammonia and day (Figure 3). As a constrained linear ordination method, RDA was used to explain the relationship between response variables (here: Hellinger-transformed OTU counts) and explanatory variables (here: treatment condition). The overall variance explained by the RDA model was 34.1% for the bacterial and 13.4% for the archaeal dataset. Analysis of variance (ANOVA) confirmed a significant interaction effect “ammonia:day” on the observed community composition.

The RDA ordination plot of the bacterial community shows very similar community composition in ammonia-treated (yellow orange) and control (blue) reactors at the start of the incubation period. This was to be expected given the identical starting material (Figure 3A). Dissimilarity in community composition between the ammonia-treated and control reactors increased with incubation time, indicating the significant interaction effect of ammonia and incubation time on bacterial community composition in the model reactors. In other words, the effect of incubation time on community composition differs depending on ammonia concentration. Changes in community composition over time in the ammonia-stressed reactors are far more pronounced compared to those in the control reactors. They lead to a completely different community composition at the end of the experiment. This clearly shows the influence of excess ammonia and the reaction of the microbial community. Analysis of variance (ANOVA) confirmed statistical significance of the temporal effect for both bacterial [*F*(1,76) = 7.15; *p* = 0.001] and archaeal community composition [*F*(1,76) = 2.75; *p* = 0.032] in the ammonia-treated reactors, although this effect was smaller in the latter (as indicated by lower explained variance).

The 10 OTUs contributing most to the explained variance of the RDA model are indicated as vectors. As shown in the ordination plot, presence of RFN20 (Erysipelotrichia) and *Acholeplasma* appears to be indicative for control reactors (already adapted to higher ammonia), while communities in additional ammonia-treated reactors are characterized by the presence of Porphyromonadaceae (Figure 3A). Based on these analyses, *Acholeplasma* and RFN20 can be seen as potential indicator organisms for stable biogas reactor communities but already adapted to higher ammonia. They could play a role in future detection of reactor imbalance due to drastic increase in ammonia and allow the initiation of earlier counteractions. However, since another recent study showed that *Acholeplasma* were identified as positively correlating with increasing ammonia concentrations and dominance of syntrophic acetate oxidation pathway (Müller et al., 2016), further studies are required also addressing the high variability of microbiomes of stable and healthy reactors.

OTUs classified as belonging to MBA08 (Clostridia) were more abundant in the beginning compared to the end of the experiment (Figure 2A). Besides the taxonomically annotated OTUs, unclassified OTU 8 was characteristic for ammonia-shocked reactors at the end of the incubation phase. The sequence showed low similarity [81.0% sequence similarity, SILVA database (v132) (Pruesse et al., 2012)] to *Solibacillus isronensis* B3W22 sequences, a strain belonging to the class Bacilli. Those are also known facultatively anaerobic degraders of polysaccharides and were reported to be present in other biogas reactors investigated (Ghosh et al., 2015). Unclassified OTU 9 showed low similarity to taxonomically annotated sequences and was most abundant in control reactors at later incubation time points (Supplementary Figure S1). The sequence of OTU 9 was most similar [85.4% sequence similarity, SILVA database (v132) (Pruesse et al., 2012)] to a sequence of a *Hydrogenoanaerobacterium saccharovorans*, a bacterium isolated from an up-flow anaerobic sludge blanket reactor which was capable of saccharide fermentation with the production of ethanol, acetate, hydrogen, and carbon dioxide (Song and Dong, 2009).

In comparison to the bacterial community, compositional differences between archaeal communities in ammonia-treated and control reactors were less pronounced, but significant nonetheless (Figure 3 and Supplementary Figure S2). A reason for the low diversity in the archaeal community is the already relatively high ammonia concentration in the used inoculum. Especially methanogenic archaea belonging to the Methanosarcinales have been observed to be sensitive toward increased ammonia concentrations (Fotidis et al., 2014) and were low in abundance at the start of the experiment. A clear association between OTU 4, classified as Vadin CA 11 (an uncharacterized archaeon belonging to the Methanomassiliicoccaceae) and control reactor samples was observed in RDA. This genus was initially identified by a partial 16S rRNA gene sequence within an anaerobic digester fed with distillery waste (Godon et al., 1997). OTU 3, classified as *Methanomicrococcus* (Methanosarcinaceae), was more abundant in samples of both control and treatment reactors at the beginning of the experiment. As OTU 1, classified as *Methanoculleus* (Methanomicrobiaceae), a methanogen frequently observed in biogas reactor systems with increased ammonia concentrations (Fotidis et al., 2014; Moestedt et al., 2016; Maus et al., 2016b), was equally dominant in the control and treatment reactors, it, therefore, did not contribute information to the differentiation between control and treatment archaeal community.

While the results of the 16S rRNA gene-based community analysis and the metatranscriptomic analysis are quite coherent in terms of community composition, it must be mentioned that potentially DNA-based approaches might not be able to completely resolve the shift of the active community within a short timeframe, such as chosen for the presented experiment. Therefore, the community composition as observed by 16S rRNA gene amplicon analysis might include inactive or dead cells, which’s DNA is still detected with this approach. This bias in terms of active and productive organisms within the biogas reactor microbiome under different conditions can be overcome by looking at the RNA level (Supplementary Figure S6).

### Metatranscriptomic Analysis Shows Reduced Transcription of Cellulases, Heterodisulfide Reductase, and Methyl-Coenzyme M Reductase Under Increased Ammonia Concentrations

Besides the taxonomic composition of the biogas reactor community, an analysis of the transcript level was subsequently performed to investigate the activity of degradation pathways and the active microbial community under treatment and control conditions.

To gain information on the genes transcribed in the studied environment, two sampling points in the experiment were selected for generation of metatranscriptomic sequences. On day 3, the highest CH_4_ production of the entire experiment was observed by GC analysis, whereas from day 6 we observed steady CH_4_ production in ammonia-treated and control reactors at different levels (Figure 1A). We particularly focused on bacterial enzymes involved in cellulose and peptide degradation, the initial steps in anaerobic degradation of organic macromolecules. An additional focus was on the archaeal enzymes of the methanogenic pathways. For this detailed analysis, metatranscriptomic reads were assembled into contigs. To increase contig length and hence improve taxonomic and functional annotation, we included publicly available metagenomic reads from the biogas reactor used as inoculum in the assembly (Güllert et al., 2016). The latter were screened for potential open reading frames (ORFs), which were further annotated using the Pfam and KEGG databases as described in the methods section. Enzyme commission numbers (E.C. numbers) were used to select key enzymes for the investigated pathways (Luo et al., 2016).

**Figure 4:**
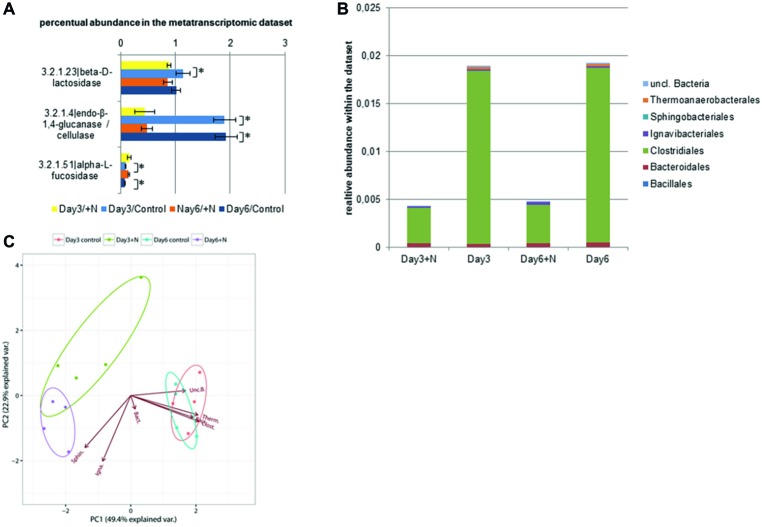
Transcript level of annotated cellulase sequences (E.C.: 3.2.1.x) is shown in section **(A)**. **(B)** Taxonomic composition of the contigs with annotated cellulase (E.C. 3.2.1.4) within the metatranscriptomic data. **(C)** PCA of square root-transformed taxonomic composition data with annotated cellulose. (Bacillales, Baci.; Bacteroidales, Bact.; Clostridiales, Clost.; Ignavibacteriales, Igna.; Sphingobacteriales, Sphin.; Thermoanaerobacterales, Therm.; unclassified Bacteria., Unc.B.).

Within the peptidases (E.C. 3.4.x.x), only one serine peptidase type (E.C. 3.4.16.4) was significantly downregulated under increased ammonia conditions (Supplementary Figure S3). In contrast, multiple carbohydrate or glycosyl hydrolases (E.C. 3.2.1.x) were affected by increased ammonia concentration (Figure 4). Especially more abundant (1–0.1% of the annotated transcripts) cellulase (β-1,4-endoglucan hydrolase; E.C. 3.2.1.4) and β-galactosidase (E.C. 3.2.1.23) showed significantly lower transcript levels under increased ammonia in the dataset while the transcript level of α-fucosidases (E.C. 3.2.1.51) was upregulated. α-Fucosidase transcript levels were however significantly lower than the transcript levels of the two aforementioned hydrogenases. Most of the transcripts coding for cellulases were annotated as clostridial sequences, followed by Bacteroidales. Interestingly, while the transcript level of cellulases from Bacteroidales remained relatively stable under ammonia stress, a clear reduction of Clostridia-affiliated sequences was observed from 95 to 82% of overall sequences. Overall cellulase transcript levels were significantly reduced at both sampling time points (Wilcoxon rank sum test: *W* = 16, *p* < 0.03). These results indicate that initial cellulose hydrolysis is reduced due to a lack of clostridial cellulases (Figure 4). This strong effect on clostridial transcripts can also be observed in the principle component analysis (PCA) based on taxonomic annotation and abundance of the transcribed cellulases. The PCA shows that the activity of Thermoanaerobacterales, Clostridiales, and Bacillales was characteristic for the control reactors, hence with a higher transcript level of genes coding for cellulases. The lower transcript level of cellulases in the ammonia-treated reactors might thus drastically affect the process of anaerobic biomass degradation. Similar effects of increased ammonia concentrations on the activity of the acetogens and the previous degradation steps have been observed by Gallert et al. (1998) and were classified as negative feedback to the removal of hydrogen by methanogens (Gallert et al., 1998).

**Figure 5:**
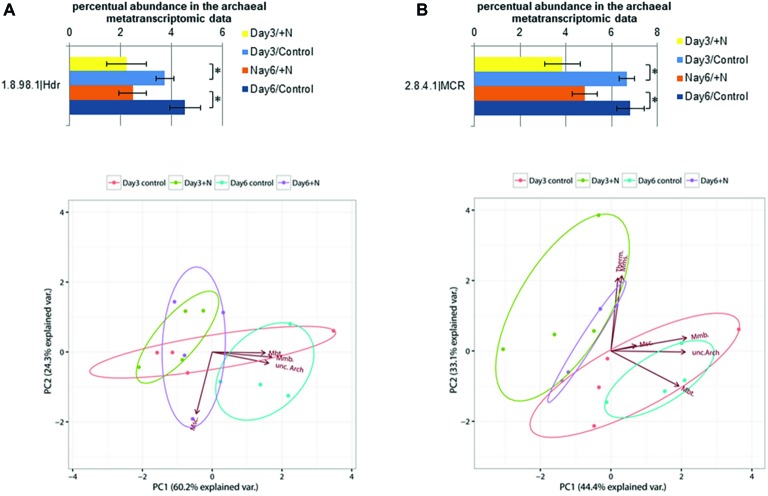
Transcript level of annotated heterodisulfide reductase sequences (E.C.: 1.8.98.1) is shown in section **(A)** and for methyl-CoM reductase sequences (E.C.: 2.8.4.1) in section **(B)**. Error bars indicate standard deviation observed between the *n* = 4 replicates per treatment and sampling point. Significance was determined by two-sided Wilcoxon-Mann-Whitney rank sum test. ^*^(*p* ≤ 0.05). PCA below was generated based on the square root-transformed taxonomic composition data of the contigs containing the corresponding enzyme (Methanobacteriales, Mbt.; Methanomicrobiales, Mmb.; Methanosarcinales, Msc.; Methanomassiliicoccales, Mms.; Thermoplasmatales, Thermo.; unclassified Archaea, unc.Arch.).

Among genes involved in the methanogenic pathway (Supplementary Figure S4), heterodisulfide reductase (Hdr, E.C. 1.8.98.1) was significantly reduced on the transcript level (Wilcoxon rank sum test: *W* = 16, *p* > 0.03) on day 6 under increased ammonia conditions (Figure 5). This enzyme catalyzes a key step in the methanogenic metabolism, the reduction of heterodisulfide (CoM-S-S-CoB) into coenzymes CoM-SH and CoB-SH (Hedderich and Thauer, 1988). Taxonomic annotation of sequences coding for Hdr showed high abundance of Methanomicrobiales (89–92%) as well as minor amounts of sequences annotated as Methanobacteriales (1–5%) and Methanosarcinales (0.4–0.6%) along with some unannotated archaeal taxa (4–6%). The abundance of the transcripts for the Hdr subunits differed between the cytosolic (*hdr*ABC) and membrane-bound (*hdr*DE) types. The membrane-bound type consisting of the two subunits HdrDE is exclusively present in methanogens of the Methanosarcinales (Thauer et al., 2008; Buan and Metcalf, 2010; Rother, 2010). While more than 94% of all found transcripts classified as Hdr were mapped onto *hdr*ABC subunit genes, *hdr*DE subunits were of extremely low abundance. In multiple samples, *hdr*E subunit transcripts were below the detection limit. The low abundance of these transcripts in addition to the low abundance of Methanomicrococcus (*Methanosarcinales*) in the 16S rRNA gene dataset further underlines the low contribution of acetoclastic methanogenesis to overall methane production in this reactor setup.

### Syntrophic Acetate Oxidizing Bacteria Are Important in Biogas Reactor Systems Lacking Acetoclastic Methanogens Like Methanosarcinaceae and Methanosaetaceae

The syntrophic acetate oxidizing bacteria are a taxonomically diverse group which transforms acetate into CO_2_ and H_2_, an overall energetically unfavorable reaction (ΔG0′ = +95 kJ/mol). Due to a syntrophic interaction with hydrogenotrophic methanogens, this endergonic process is coupled to the exergonic methane formation (ΔG0′ = −135 kJ/mol) and the overall reaction becomes exergonic (Schink, 1997; Stams et al., 2006).

**Figure 6:**
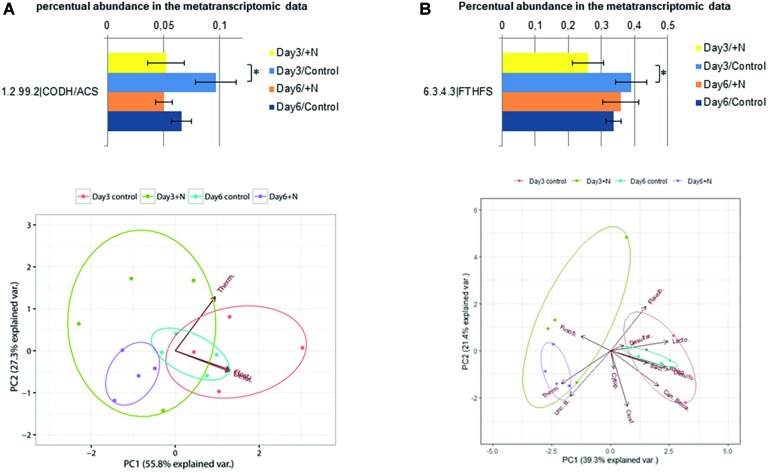
Transcriptional activity of annotated carbon monoxide dehydrogenase/acetyl CoA synthase sequences (E.C.: 1.2.99.2) is shown in section **(A)** and for formyl tetrahydrofolate synthase sequences (E.C.: 6.3.4.3) in section **(B)**. Error bars indicate standard deviation observed between the *n* = 4 replicates per treatment and sampling point. Significance was determined by two-sided Wilcoxon-Mann–Whitney rank sum test. ^*^(*p* ≤ 0.05). PCA below was generated based on the square root-transformed taxonomic composition data of the contigs containing the corresponding enzyme (Bacillales, Baci., Candidatus Brocadiales, Can. Broca., Clostridiales, Clost., Cytophagales, Cytop., Desulfarculales, Desulfar., Desulfobacterales, Desulfo., Flavobacteriales, Flavob., Fusobacteriales, Fusob., Lactobacillales, Lacto., Rhizobiales, Rhizo., Thermoanaerobacterales, Therm., uncl. Bacteria, Unc. B.).

In transcriptional studies focusing on the syntrophic acetate oxidation and acetogenic bacteria, transcription analysis of genes encoding two key enzymes has recently been applied in environmental studies, formyltetrahydrofolate synthetase (Fthfs, E.C. 6.3.4.3) and carbon monoxide dehydrogenase/acetyl-CoA synthase complex (Codh/Acs, E.C. 1.2.99.2) (Müller et al., 2013, 2016). Both enzymes are involved in the Wood-Ljungdahl pathway (WLP) and they have been applied in several studies investigating the acetogenic and syntrophic acetate oxidizing community (Leaphart and Lovell, 2001; Pester and Brune, 2006; Hori et al., 2011; Müller et al., 2013, 2016). Within the methyl branch of the WLP, the formyltetrahydrofolate synthetase is catalyzing the reaction of formate with tetrahydrofolate to formyl N-10-tetrahydrofolate, which is consecutively reduced to methyl-tetrahydrofolate. The methyl group of this compound is transferred to the carbon monoxide dehydrogenase/acetyl-CoA synthase complex bound to a corrinoid iron sulfur protein CoFeS-P (Mayer and Müller, 2014). The carbon monoxide dehydrogenase/acetyl-CoA synthase is performing the carbonyl branch of the WLP and reduces CO_2_ to CO which forms the future carboxyl group of the formed acetyl-CoA (Hattori et al., 2005; Hattori, 2008). The acetyl-CoA is subsequently phosphorylated by the phosphotransacetylase to form acetyl phosphate and then turned into acetate *via* an acetate kinase (Mayer and Müller, 2014). Since the carbonyl branch of the WLP can be bypassed through decarboxylase and glycine reductase (Ljungdahl, 1986; Leaphart and Lovell, 2001), the gene coding for the Fthfs seems to be the more reliable marker to monitor the syntrophic acetate oxidizing and acetogenic community (Müller et al., 2016). Transcript levels for both marker genes were significantly reduced on day 3 (Wilcoxon rank sum test: *W* = 16, *p* > 0.05) (Figure 6). Transcripts of the Codh/Acs marker gene were taxonomically less diverse compared to Fths transcripts and mostly assigned to the Clostridiales (36–70%), Desulfuromonadales (10–20%), and Thermoanaerobacterales (20–45%). These orders were also among the most abundant within the dataset of the Codh/Acs transcripts. The most abundant orders within the Codh/Acs dataset were the Clostridiales (33–44%), Desulfobacterales (8–31%), Cytophagales (1–10%), and Thermoanaerobacterales (2–5%). In addition, 15–37% of the transcripts were assigned as unclassified bacteria, which underlines the importance of further investigation in regards to this pathway. The abovementioned orders were all already described as part of biogas reactor microbiomes [Desulfobacterales: (Lebiocka et al., 2018), Cytophagales: (Świątczak et al., 2017), others: (Krause et al., 2008)]. Especially Clostridiales and Thermoanaerobacterales have been identified as typical syntrophic bacteria involved in SAO (Moestedt et al., 2016; Müller et al., 2016). Since the transcription of both marker genes was reduced by the initial ammonia shock on day 3, it might be possible that also the syntrophic acetate oxidizing bacteria, and, within them, especially the Thermoanaerobacterales are affected by a drastic increase in ammonia. Since this effect was not significant on day 6, Thermoanaerobacterales potentially are able to adapt to increased ammonia concentrations. In a study by Bonk et al. (2018), the effects of increased ammonia concentrations (5 g/L TAN) on the metabolization of acetate, butyrate, and propionate by syntrophic bacteria was investigated in more detail. The authors of this study observed differences in the tolerance of the different syntrophic bacteria. This study showed higher tolerance of Syntrophomonas, while Syntrophobacter decreased in abundance and was potentially replaced by so far unidentified propionate degraders under increased ammonia conditions (Bonk et al., 2018). These findings in regard to the sensitivity of the overall syntrophic community involved in the anaerobic degradation within biogas reactors need to be further investigated in future studies.

The typical marker gene of the methanogenic pathway coding for methyl-CoM reductase (Mcr, E.C. 2.8.4.1) showed higher transcript levels in control reactors (Figure 5B). While this effect was not significant for the whole experimental setup at the 5% level, it showed a tendency toward lower expression levels under increased ammonia conditions at both sampling points (Wilcoxon rank sum test: *W* = 12, *p* = 0.343). The methyl-CoM reductase is involved in the reduction of the methyl group bound to CoM-SH in the final step of methanogenesis and release of CH_4_ (Gunsalus and Wolfe, 1980; Thauer et al., 2008). The transcript level of genes encoding Mcr has been demonstrated to be reduced under increased ammonia concentration in anaerobic digester sludge (Zhang et al., 2014; Lv et al., 2019). Most of the transcripts for Mcr were taxonomically affiliated with Methanomicrobiales (37–45%), Methanosarcinales (9–17%), Methanomassiliicoccales (5–11%), and Methanobacteriales (3–9%). In addition, 3–8% of the sequences were affiliated with Thermoplasmatales. This order is closely related to the methanogenic Methanoplasmatales, which were recently isolated from termite guts (Paul et al., 2012). A noticeable proportion of sequences (26–34%) functionally annotated as coding for Mcr were not taxonomically assignable, which shows the need for further investigation of the archaeal and methanogenic community.

Transcription patterns of genes encoding enzymes involved in methanogenesis were generally comparable with community abundance patterns inferred by 16S rRNA gene amplicon analysis (Supplementary Figure S5). Taxonomic classification of the transcripts indicated Methanomicrobiales to be the dominant order involved in methane production and therefore suggests higher activity of the hydrogenotrophic methanogenesis pathway. However, transcripts of Methanomicrobiales were less abundant than 16S rRNA genes of this order. In general, Methanobacteriales and Methanosarcinales are both capable of hydrogenotrophic methanogenesis.

**Figure 7:**
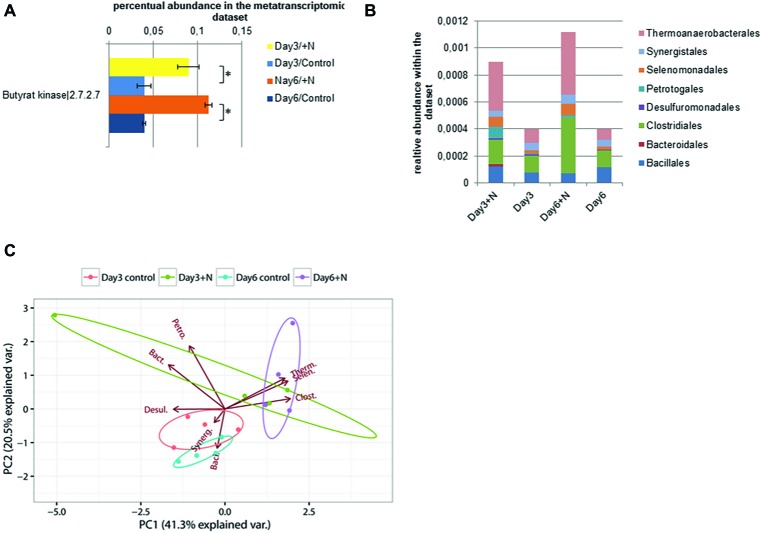
Transcript level of annotated butyrate kinase sequences (E.C.: 2.7.2.7) is shown in section **(A)**. **(B)** Taxonomic composition of the contigs with annotated butyrate kinase within the metatranscriptomic data. **(C)** PCA of square root-transformed taxonomic composition data with annotated butyrate kinase (Bacillales, Baci.; Bacteroidales, Bact.; Clostridiales, Clost.; Desulfuromonadales, Desul.; Petrotogales, Petro.; Selenomonadales, Selen.; Synergistales, Synerg.; Thermoanaerobacterales, Therm.).

The reduction in transcript levels of key genes for methanogenesis further correlated with the observed reduction in methane production in ammonia-treated reactors. Since the methanogenic pathway was significantly inhibited by increased ammonia concentrations, usually an increase in the concentration of VFAs can be observed (Chen et al., 2016; Shi et al., 2016). Even though an increase in the concentrations of acetate and butyrate was observed under increased ammonia conditions, we did not observe a decrease in pH. The pH remained relatively stable during the experiment (Figure 1A), caused by the carbonate/bicarbonate buffer present in the environment (25.7 g CaCO_3_/L total alkalinity of carbonate). Under increased ammonia concentration, an upregulation of genes involved in butyrate formation was observed in the later sampling time points. Among them were the genes coding for butyrate kinase (Buk, E.C. 2.7.2.7) and for phosphotransbutyrylase (Ptb, E.C. 2.3.1.19). These showed significant upregulation under increased ammonia concentration at later time points (Figure 7). Taxonomic analysis showed that under increased ammonia condition, especially Thermoanaerobacteriales (39–42%), Clostridiales (19–37%), and Selenomonadales (8%), all belonging to Firmicutes, contribute to increased transcript level of the aforementioned genes.

Overall, the observed taxonomic composition and transcriptional profile were typical for a mesophilic biogas reactor (Zakrzewski et al., 2012; Güllert et al., 2016). Taxonomic and functional analyses provided evidence for dominance of hydrogenotrophic methanogenesis in the tested reactor setup. In the initial step of anaerobic degradation, substrate hydrolysis, minor effects of increased ammonia levels on peptidases were observed. However, mainly strong and significant effects on cellulase transcript levels (endo-β-1,4-glucanase/cellulase, E.C. 3.2.1.4) were observed. Regarding the composition of taxa, we detected a strong impact of increased ammonia on transcripts affiliated with Clostridiales, which were identified as the main contributors to cellulose and polysaccharide degradation in biogas reactors (Güllert et al., 2016). This finding points toward a strong inhibition of anaerobic degradation already at an early step of the degradation (hydrolysis stage). If this is a direct effect of increased ammonia levels on the cellulolytic microorganisms, a feedback from accumulating products like butyrate and acetate or a combination of both needs to be investigated in future experiments. In addition, a significant reduction of gene transcripts encoding enzymes involved in methanogenesis was observed. This observation was consistent with reduced methane production under increased ammonia concentrations. Notably, we observed a stronger effect on transcript level of the archaeal heterodisulfide reductase genes compared to the usually applied marker gene for methanogenesis, methyl-CoM reductase. This higher sensitivity of transcription of genes coding for the archaeal Hdr could be beneficial for the application as an early indicator for reactor fitness either on the transcript level as presented here or by measurement of the enzyme’s activity in the environment.

## Conclusion

The microbial community developed differently over time on both the control and ammonia-treated reactors. Changes in the bacterial community were more pronounced compared to the less diverse archaeal community. A significant reduction of several bacterial families like Acholeplasmataceae and Erysipelotrichaceae was observed with increased ammonia levels. Consequently, these families are attractive candidates for the use as indicator taxa for process imbalance in the tested reactor setup. The most abundant classes throughout the experiment were Clostridia and Bacteroidia. Under increased ammonia conditions, the transcript level of cellulase-coding genes was significantly reduced. Taxonomic annotation of observed cellulase-coding genes supported the picture of Clostridia as main hydrolytic class in the biogas reactor environment (Güllert et al., 2016). Clostridia harbor a versatile enzymatic complex for cellulose degradation, the cellulosome (Lynd et al., 2002). Bacteroidial cellulases were observed but found to be less abundant under both control and ammonia-treated conditions, even though this class competes with Clostridia in other environments and under different conditions (Güllert et al., 2016). Dominance of the genus *Methanoculleus* based on 16S rRNA gene amplicon sequencing was observed during the entire experiment. This indicated a predominance of hydrogenotrophic methanogenesis coupled to syntrophic acetate oxidation in this biogas reactor setup, which is in accordance with previous observations in biogas reactors under increased ammonia concentrations (Schnürer et al., 1999; Schnürer and Nordberg, 2008; Poirier et al., 2016).

High transcript levels of Methanomicrobiales methyl-CoM reductase and heterodisulfide reductase genes further supported a dominance of hydrogenotrophic methanogenesis. The observed sensitivity of the heterodisulfide reductase transcription may be an alternative or addition to the already established marker gene *mcrA*. Additional experiments with labeled acetate (Fotidis et al., 2014) or proteomic investigation of presence or activity of identified key enzymes (Heyer et al., 2013, 2015) are required for a final validation of this speculation and to determine the fate of acetate in the biogas reactors. Nonetheless, our results strongly indicate dominance of hydrogenotrophic methanogenesis for methane production in the studied setup simulating a drastic increase of ammonium levels in a biogas reactor.

## Data Availability

Sequencing data were submitted to NCBI, accession number: SRX3323391 to SRX3323566.

## Author Contributions

MF, RS, and KW designed the experiment. MF, KW, SR, and AU performed the experiments. MF and AU extracted nucleic acids and prepared 16S amplicon libraries. Sequencing of the 16S amplicon pools as well as rRNA depletion and preparation of metatranscriptomic libraries and sequencing was performed by SK at the MPI Plön. Data were bioinformatically analyzed by SN and MF. MF, SN, SR, and RS interpreted the data. MF wrote the manuscript with input from all authors. All authors approved the final version of the manuscript.

### Conflict of Interest Statement

The authors declare that the research was conducted in the absence of any commercial or financial relationships that could be construed as a potential conflict of interest.
