# Supplementary material for: Immediate Effects of Ammonia Shock on Transcription and Composition of a Biogas Reactor Microbiome
Source: Front Microbiol. 2019 Sep 6;10:2064. doi: 10.3389/fmicb.2019.02064 (PMC6742706; doi:10.3389/fmicb.2019.02064)
Supplement: Supplementary file 1 [file Data_Sheet_1.pdf]

Supplemental material

Table S1: Sequences of the primers used for 16S rRNA gene amplicon sequencing of the bacterial and archaeal domain. Primer sequence consisted of an initial standardized Illumina adapter (regular), followed by an 8 nucleotide barcode (X's) and a primer sequence (**bold**). Additionally, primers for the archaeal 16S analysis contained a linker sequence for the sequencing reaction on the flow cell (<u>underlined</u>).

	Primer name	Sequence of the primers
Archaea	Fwd Ar0787	AATGATACGGCGACCACCGAGATCTACAC XXXXXXXX <u>ACACTCTTTCCCTACACGACGCTCTTCCGATCT</u> **ATTAGATACCCSBGTAGTCC**
	Rev Ar1059	CAAGCAGAAGACGGCATACGAGAT XXXXXXXX <u>GTGACTGGAGTTCAGACGTGTGCTCTTCCGATCT</u> **GCCATGCACCWCCTCT**
Bacteria	Fwd Ba0027	AATGATACGGCGACCACCGAGATCTACAC XXXXXXXX **TATGGTAATTGTAGAGTTTGATCCTGGCTCAG**
	Rev Ba0338	CAAGCAGAAGACGGCATACGAGAT XXXXXXXX **AGTCAGTCAGCCTGCTGCCTCCCGTAGGAGT**

Table S2: Summary of the raw and filtered reads used for the assembly and analysis of the metatranscriptomic dataset.

	raw reads	filtered reads
Reactor1-day3(+Ammonia)	5596956	5118144
Reactor2-day3(+Ammonia)	7445220	7128868
Reactor3-day3(+Ammonia)	7762252	6811958
Reactor4-day3(+Ammonia)	8043546	7464842
Reactor5-day3(+Control)	6803484	6825148
Reactor6-day3(+Control)	6794676	6126394
Reactor7-day3(+Control)	6754515	5473714
Reactor8-day3(+Control)	9928674	9362436

Table S3: Summary of the raw and filtered reads generated via 16S rRNA gene amplicon sequencing.

	raw sequences	filtered sequences
Bacteria	15054122	5830548
Archaea	105018664	8250620

Table S4: 16S sequence counts per individual sample used in the 16S rRNA gene amplicon analysis. Reactors 1-4 correspond to the ammonia treatment, reactors 5-8 correspond to the control.

	Bacteria	Archaea
Reactor 1 Day 1	130435	23507
Reactor 1 Day 2	73331	16970
Reactor 1 Day 3	2192	65682
Reactor 1 Day 4	2453	99418
Reactor 1 Day 5	2434	83894
Reactor 1 Day 6	144511	115805
Reactor 1 Day 7	80899	70497
Reactor 1 Day 8	2182	58522
Reactor 1 Day 9	3311	47263
Reactor 1 Day 10	2986	74835
Reactor 2 Day 1	2327	19257
Reactor 2 Day 2	1743	27021
Reactor 2 Day 3	2166	19473
Reactor 2 Day 4	99910	50410
Reactor 2 Day 5	3017	66825
Reactor 2 Day 6	2491	72680
Reactor 2 Day 7	84768	106396
Reactor 2 Day 8	60157	47569
Reactor 2 Day 9	3211	43471
Reactor 2 Day 10	2955	46529
Reactor 3 Day 1	77004	16086
Reactor 3 Day 2	1864	27113
Reactor 3 Day 3	2191	20491
Reactor 3 Day 4	1964	76755
Reactor 3 Day 5	2047	59829
Reactor 3 Day 6	75195	48699
Reactor 3 Day 7	76127	79443
Reactor 3 Day 8	2454	39040
Reactor 3 Day 9	108167	76646
Reactor 3 Day 10	95623	52076
Reactor 4 Day 1	2611	58623
Reactor 4 Day 2	87248	14732
Reactor 4 Day 3	2673	19400
Reactor 4 Day 4	2599	105107
Reactor 4 Day 5	87679	90098
Reactor 4 Day 6	61889	80034
Reactor 4 Day 7	62674	79750
Reactor 4 Day 8	94261	114734
Reactor 4 Day 9	3504	51412

Reactor 4 Day 10	81915	25769
Reactor 5 Day 1	2303	37661
Reactor 5 Day 2	1930	32244
Reactor 5 Day 3	2150	22913
Reactor 5 Day 4	1773	90998
Reactor 5 Day 5	2336	42299
Reactor 5 Day 6	2613	50335
Reactor 5 Day 7	75643	59434
Reactor 5 Day 8	85052	64425
Reactor 5 Day 9	2868	65723
Reactor 5 Day 10	2651	75034
Reactor 6 Day 1	2545	27022
Reactor 6 Day 2	3264	27557
Reactor 6 Day 3	2977	46664
Reactor 6 Day 4	64461	106528
Reactor 6 Day 5	2143	86737
Reactor 6 Day 6	2322	71533
Reactor 6 Day 7	2062	35799
Reactor 6 Day 8	81832	58932
Reactor 6 Day 9	78836	31949
Reactor 6 Day 10	2910	30876
Reactor 7 Day 1	74497	22272
Reactor 7 Day 2	52235	20056
Reactor 7 Day 3	1744	96582
Reactor 7 Day 4	2139	113504
Reactor 7 Day 5	2774	88870
Reactor 7 Day 6	2708	82054
Reactor 7 Day 7	2518	72828
Reactor 7 Day 8	2756	95701
Reactor 7 Day 9	93109	69076
Reactor 7 Day 10	91344	21627
Reactor 8 Day 1	1557	21907
Reactor 8 Day 2	2409	35231
Reactor 8 Day 3	82634	70513
Reactor 8 Day 4	1828	86523
Reactor 8 Day 5	2425	52371
Reactor 8 Day 6	63780	53478
Reactor 8 Day 7	96270	57077
Reactor 8 Day 8	128246	54608
Reactor 8 Day 9	1972	43765
Reactor 8 Day 10	77114	26599

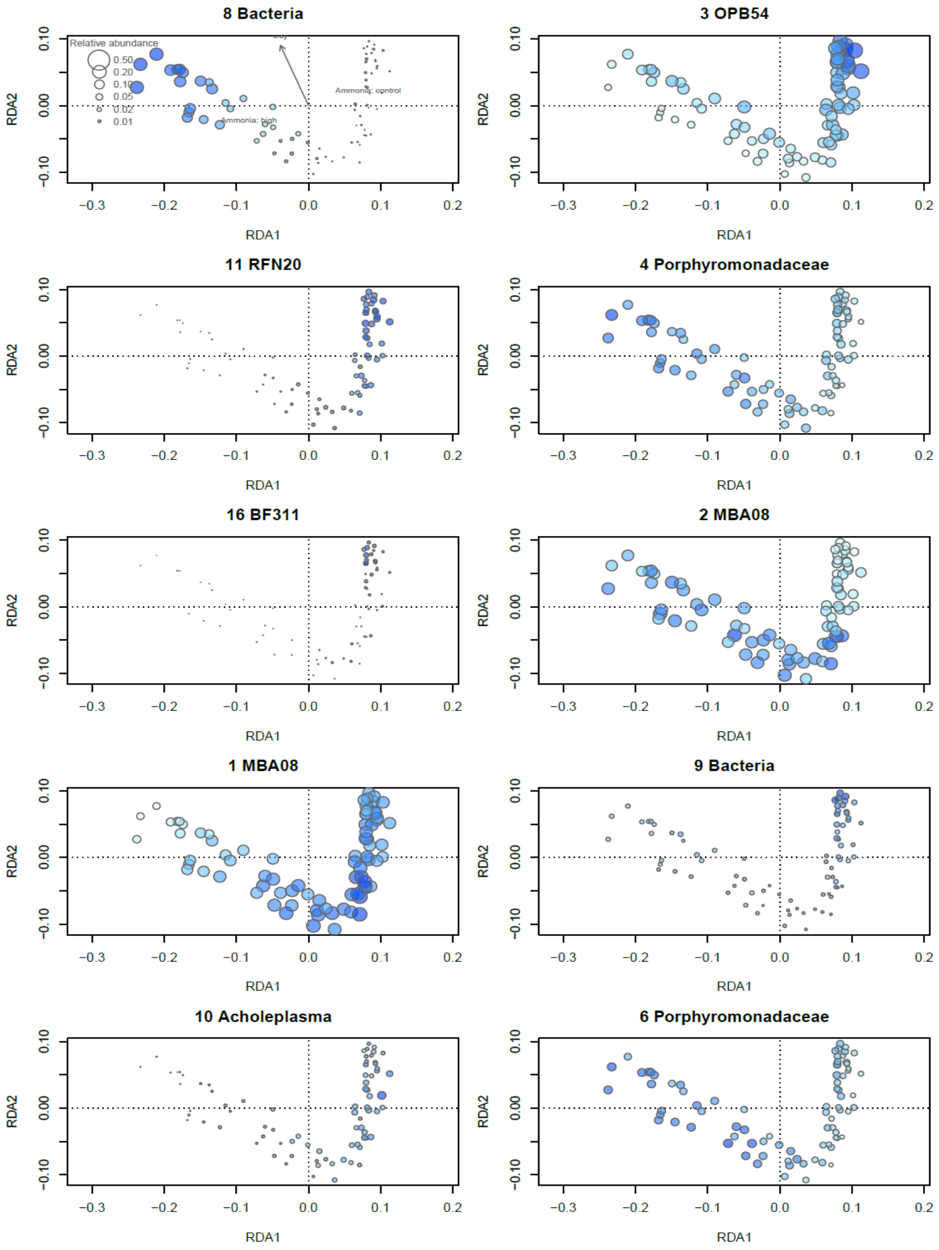

Figure S1: Distribution of the 10 bacterial OTUs contributing the most variance to the RDA within the reactor samples. Size and hue intensity of the dots correlate with the overall abundance of the respective OTU in the reactor sample.

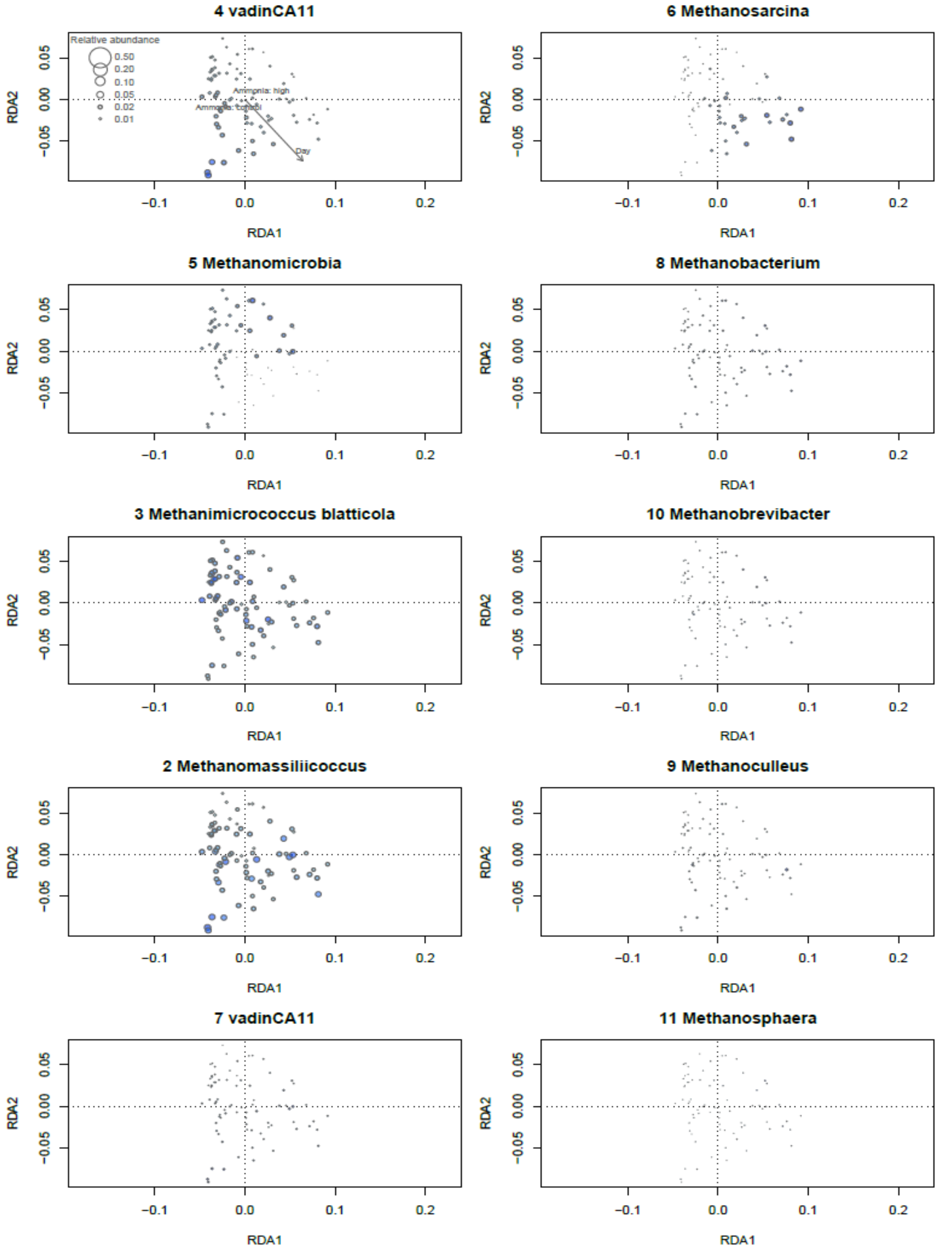

Figure S2: Distribution of the 10 archaeal OTUs contributing the most variance to the RDA within the reactor samples. Size and hue intensity of the dots correlate with the overall abundance of the respective OTU in the reactor sample.

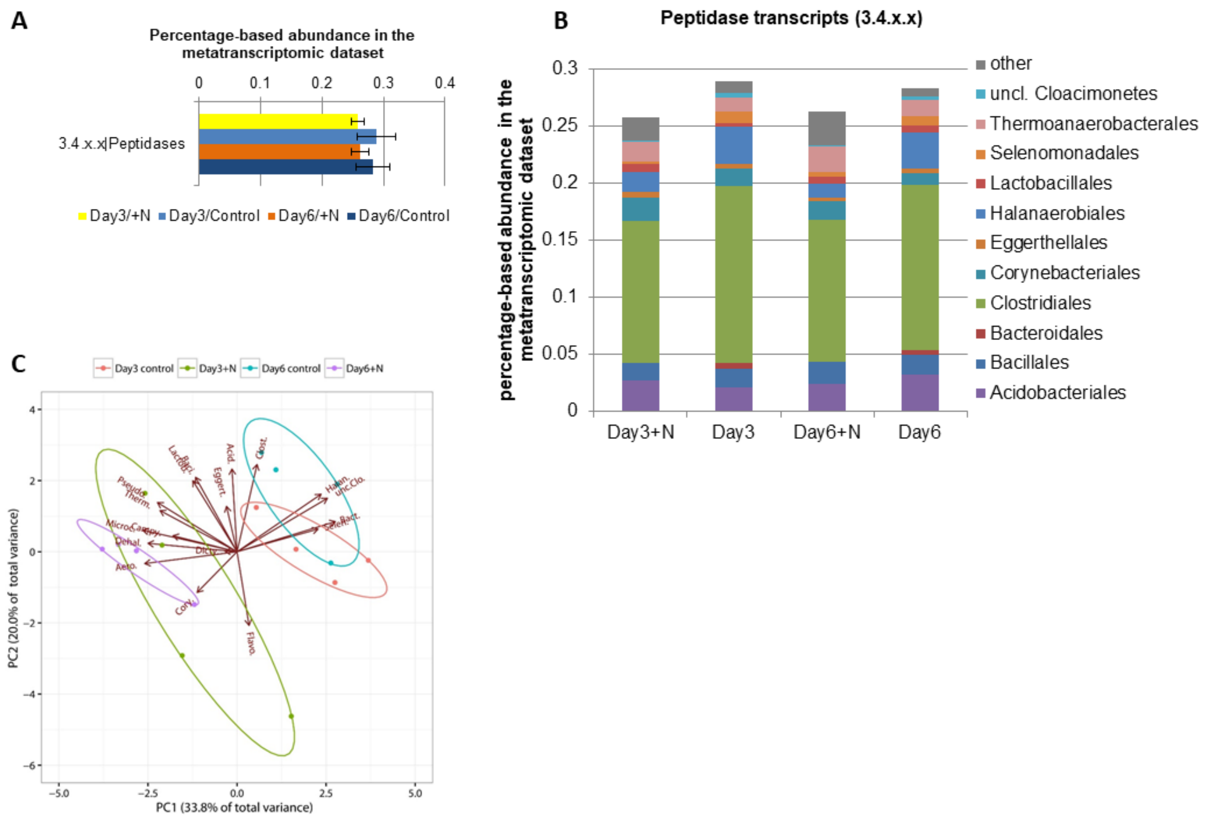

Figure S3: Transcript level of annotated peptidase sequences (E.C.: 3.4.x.x) is show in section (A). (B) Taxonomic composition of the contigs with annotated peptidases within the metatranscriptomic data. (C) PCA of square root transformed taxonomic composition data with annotated peptidases. (Acidobacteriales=Acid.; Aeromonadales=Aero.; Bacillales=Baci.; Bacteroidales=Bact.; Campylobacterales=Campy.; Clostridiales=Clost.; Corynebacteriales=Cory.; Dehalococcoidales=Dehal.; Dictyoglomales=Dicty.; Eggerthellales=Eggert.; Flavobacteriales=Flavo.; Halanaerobiales=Halan.; Lactobacillales=Lactob.; Micrococcales=Microc.; Pseudonocardiales=Pseudo.; Selenomonadales=Selen.; Thermoanaerobacterales=Therm.; uncl. Cloacimonetes=unc.Clo.).

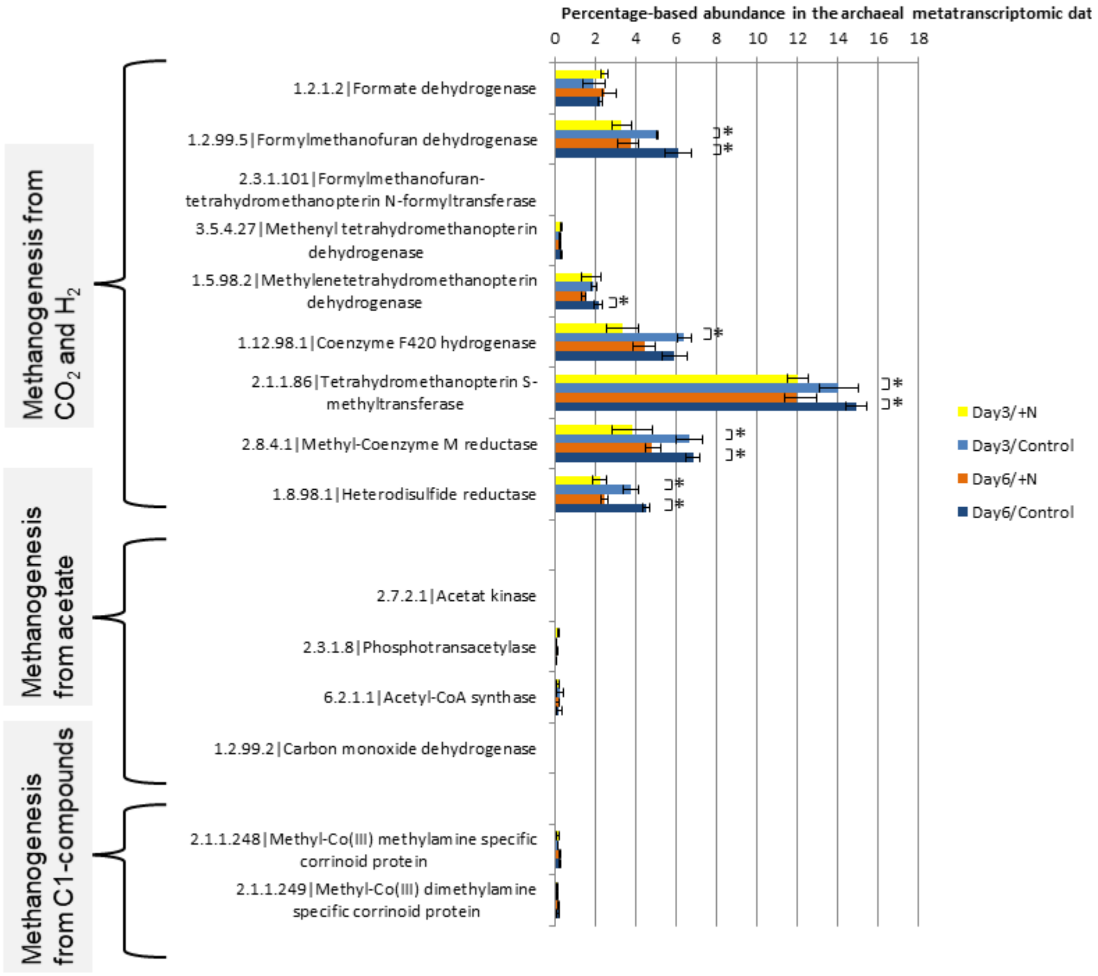

Figure S4: Transcript level of enzymes involved in the methanogenesis pathway. Significance values for the transcriptional activity was calculated by Wilcoxon rank sum test. Error bars indicate standard deviation observed between the n=4 replicates per treatment and sampling point. Significance was determined by two-sided Wilcoxon-Mann-Whitney rank sum test. *($p \leq 0.05$).

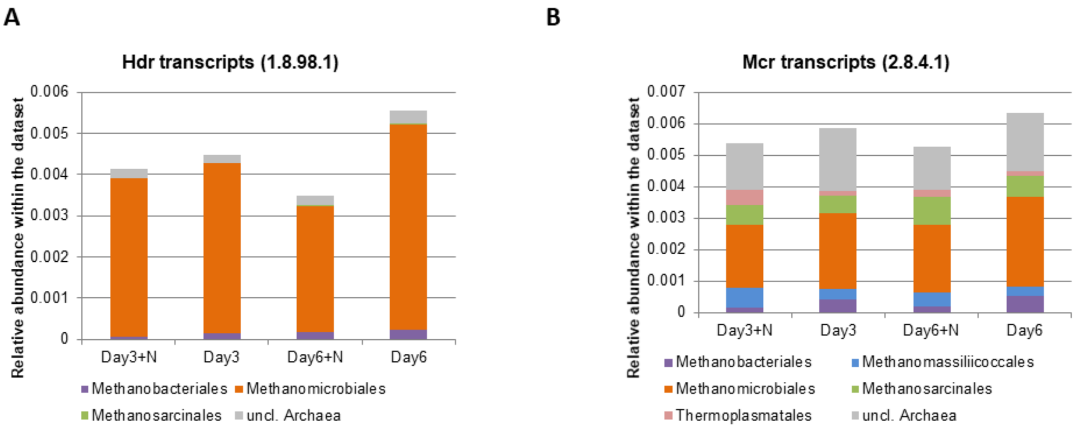

Figure S5: (A) Taxonomic composition of the contigs with annotated heterodisulfide reductase within the metatranscriptomic data. (B) Taxonomic composition of the contigs with annotated methyl-CoM reductase within the metatranscriptomic data.

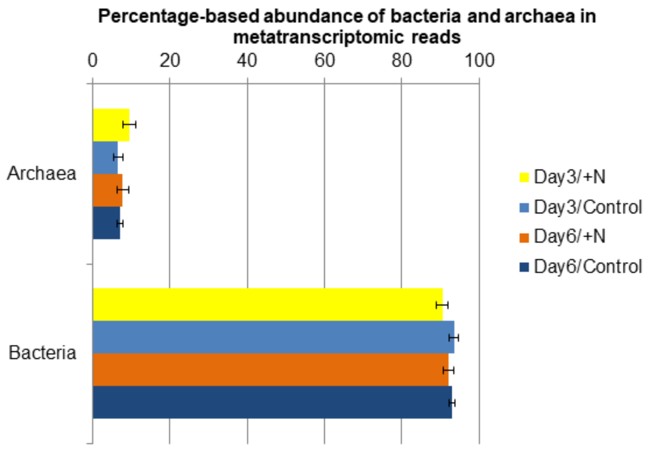

Figure S6: Archaeal and Bacterial abundance within the metatranscriptomic data at the two sampling points investigated.
